# Aggressive leiomyosarcoma of the oral cavity: a rare case report

**DOI:** 10.1093/jscr/rjaf404

**Published:** 2025-06-16

**Authors:** Amine Oussalem, Bouchra Dani, Malik Boulaadas

**Affiliations:** Department of Maxillo-facial Surgery, Specialty Hospital, Area Lamfadel Cherkaoui, Rabat - Institut B.P. 6527, Rabat 10060, Morocco; Department of Maxillo-facial Surgery, Specialty Hospital, Area Lamfadel Cherkaoui, Rabat - Institut B.P. 6527, Rabat 10060, Morocco; Department of Maxillo-facial Surgery, Specialty Hospital, Area Lamfadel Cherkaoui, Rabat - Institut B.P. 6527, Rabat 10060, Morocco

**Keywords:** oral neoplasms, leiomyosarcoma, gingival neoplasms, rare surgical case, case report

## Abstract

Leiomyosarcoma is a rare malignant tumor of smooth muscle, typically found in the uterus and gastrointestinal tract. Its occurrence in the oral cavity is extremely rare due to limited smooth muscle presence. Diagnosis relies on histopathology, and treatment involves wide surgical resection with adjuvant therapy. A 41-year-old male presented with a progressive gingivomandibular swelling for 1 year. He had previously undergone excision of a gingival lesion diagnosed as leiomyoma, which recurred after 1 year. Examination revealed a firm, non-bleeding mass from teeth 37 to 44, with hypoesthesia in the left labiomental region and mobility of the incisivocanine complex. Imaging showed aggressive features, including cortical bone lysis at the symphysis, suggesting malignancy. A biopsy confirmed leiomyosarcoma, and further imaging ruled out metastases or another primary tumor. This case underscores the importance of early diagnosis and aggressive treatment for optimal prognosis.

## Introduction

Leiomyosarcoma is a malignant tumor of smooth muscle, commonly found in the uterus, gastrointestinal tract, and blood vessels [1]. Its occurrence in the oral cavity is extremely rare due to minimal smooth muscle presence [2]. Diagnosing oral leiomyosarcoma is challenging, requiring differentiation from benign lesions like leiomyomas [3, 4].

Smooth muscle tumors in the oral region are typically benign, but malignancy must be considered when clinical suspicion arises. Due to its rarity, oral leiomyosarcoma is often overlooked or misdiagnosed, delaying appropriate treatment [5, 6]. Early detection is crucial, as management involves wide surgical resection and potentially adjunctive therapies such as radiotherapy or chemotherapy [7, 8]. This article reviews the clinical, diagnostic, and therapeutic aspects of oral leiomyosarcoma, emphasizing the importance of maintaining a high index of suspicion for rare malignancies [2–9].

## Case report

A 41-year-old male was referred for evaluation of a progressively enlarging gingivomandibular swelling over one year. He had no significant medical, familial, or genetic history. Previously, a gingival lesion near tooth 33, diagnosed as leiomyoma, recurred 1year after excision.

**Figure 1:**
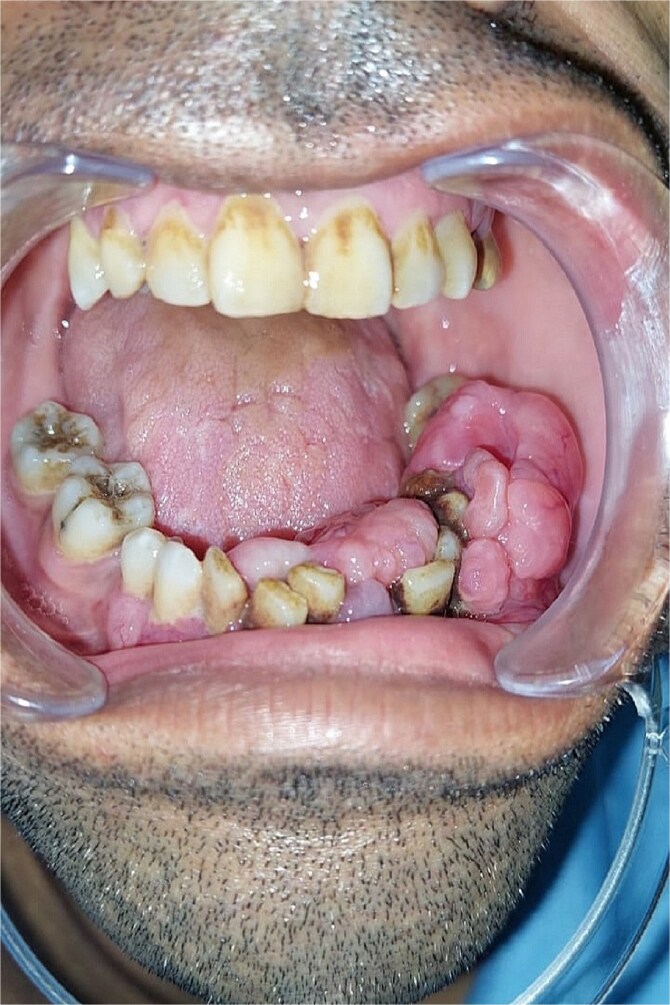
Pre-operative image showing the intraoral appearance of the leiomyosarcoma with gingival hypertrophy.

Clinical examination revealed a moderately painful, non-bleeding mass from teeth 37 to 44, with mobility of the incisivocanine complex and hypoesthesia in the left labiomental area. The lesion was localized, with no infection or acute inflammation signs (Fig. 1).

**Figure 2:**
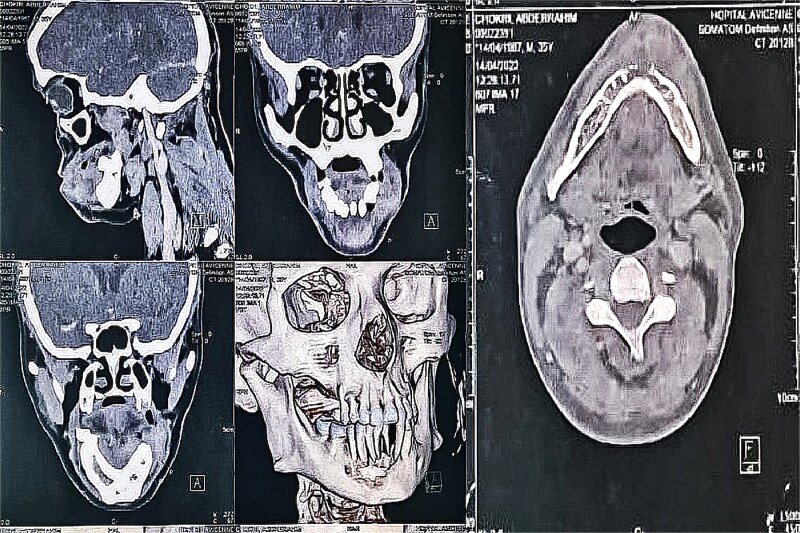
CT scan of the facial mass with axial, coronal, and sagittal slices, as well as 3D reconstructions, showing the tumor and its aggressiveness with cortical lysis at the symphysis.

Facial CT, including 3D reconstructions, showed aggressive tumor behavior with mandibular cortical lysis at the symphysis, suggesting local invasion (Fig. 2).

A biopsy confirmed leiomyosarcoma, and imaging ruled out metastasis or other tumors.

**Figure 3:**
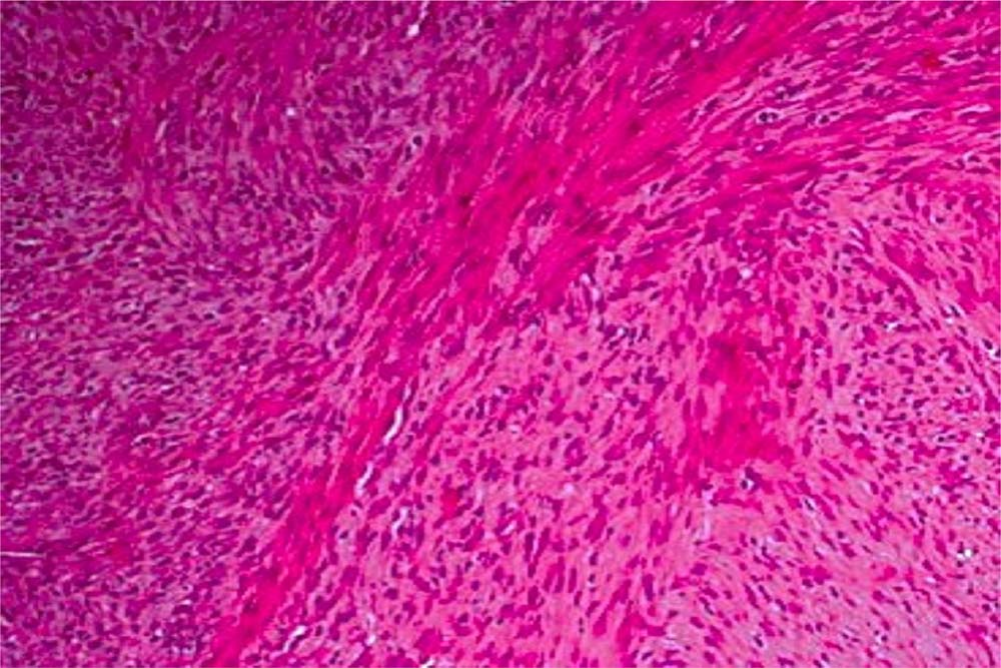
Haematoxylin and eosin stain at ×100 magnification showing a leiomyosarcoma composed of perpendicularly oriented fascicles of spindle-shaped cells.

The patient underwent wide surgical resection, including dental extractions and bone curettage (Fig. 3). He then received external beam radiotherapy (65 Gy).

At 6-month, 1-year, and 2-year follow-ups, no recurrence or metastasis was observed, highlighting the diagnostic challenge.

## Discussion

Leiomyosarcoma is an extremely rare malignancy in the oral cavity, primarily due to the limited smooth muscle presence in this region. Unlike the uterus or gastrointestinal tract, where smooth muscle tumors are more common, the oral cavity contains minimal smooth muscle, particularly in the gingiva. These tumors may arise from the smooth muscle of blood vessels, which are abundant in the oral cavity [1].

Oral leiomyosarcoma often presents as a painless lesion, leading to misdiagnosis as benign conditions like fibromas or inflammatory gingival masses, complicating early diagnosis. In our case, a recurrence of a previously excised gingival lesion diagnosed as leiomyoma, with aggressive radiologic features, raised suspicion for malignancy. This highlights the importance of histological vigilance in surgical decision-making [2, 3].

Histopathologically, leiomyosarcoma is characterized by spindle-shaped cells with eosinophilic cytoplasm and elongated nuclei in fascicular patterns. Immunohistochemistry, including desmin and smooth muscle actin, helps differentiate it from other spindle-cell neoplasms [4], with calponin improving diagnostic specificity [5].

A crucial aspect of management is evaluating metastasis. Despite its aggressiveness, oral leiomyosarcoma often lacks distant spread at diagnosis, with the lungs being the most common site of metastasis [6]. In our case, imaging, including contrast-enhanced CT and 3D mandibular reconstruction, revealed no distant disease, in line with Tanaka *et al.*’s findings [7].

Surgical management involves wide resection with clear margins, which is critical for prognosis [8]. Achieving negative margins in the mandibular region can be challenging due to anatomical constraints like the inferior alveolar nerve and dental roots. Our patient underwent wide excision, including marginal mandibular osteotomy from tooth 37 to 44, along with curettage and recontouring. Intraoperative nerve preservation was attempted, though hypoesthesia persisted, a common postoperative complication in mandibular surgeries [9].

Frozen section analysis was performed intraoperatively to ensure clear margins, a step crucial for preventing recurrence in head and neck sarcomas [10]. The surgical field was extended to include the suspected zone of perineural invasion.

Adjuvant radiotherapy was administered due to the tumor’s size and histologic grade, with a total dose of 65 Gy. This approach aimed to minimize exposure to adjacent tissues like the contralateral mandible, salivary glands, and neurovascular bundles. Several studies support postoperative radiotherapy in achieving local control in sarcomas, despite limited evidence specific to leiomyosarcoma [11]. Multimodal therapy has also been shown to improve outcomes in high-grade lesions [12].

Prognosis depends on tumor characteristics and surgical execution. Early, aggressive management, including en bloc excision and intraoperative margin control, has been associated with better outcomes [13]. Our patient remains recurrence-free 2 years post-treatment, emphasizing the importance of long-term follow-up. Functionally, the patient experienced persistent hypoesthesia, with acceptable oral function and aesthetics maintained.

Given the rarity of oral leiomyosarcoma, each case offers valuable insights for refining treatment strategies in this challenging location.

## Conclusion

Oral leiomyosarcoma is a rare malignancy that necessitates high clinical suspicion in cases of persistent gingival swelling. Early diagnosis, aided by imaging, is essential for treatment planning. Wide surgical excision remains the cornerstone of therapy, with adjuvant radiotherapy considered in aggressive cases. Long-term follow-up is vital to detect recurrence and improve prognosis.
